# Arabidopsis Histone Variant H2A.X Functions in the DNA Damage-Coupling Abscisic Acid Signaling Pathway

**DOI:** 10.3390/ijms25168940

**Published:** 2024-08-16

**Authors:** Peng Guo, Tian-Jing Wang, Shuang Wang, Xiaoyuan Peng, Dae Heon Kim, Yutong Liu

**Affiliations:** 1 Key Laboratory of Molecular Epigenetics of the Ministry of Education (MOE), Northeast Normal University, Changchun 130024, China; guop806@nenu.edu.cn (P.G.); wangtj010@nenu.edu.cn (T.-J.W.); wangs540@nenu.edu.cn (S.W.); pengxy449@nenu.edu.cn (X.P.); 2 Department of Biomedical Science, Sunchon National University, Suncheon 57922, Republic of Korea

**Keywords:** *Arabidopsis thaliana*, histone variants, genotoxic stress, ABA responses, DNA damage, CRISPR/Cas9

## Abstract

Environmental variations initiate chromatin modifications, leading to the exchange of histone subunits or the repositioning of nucleosomes. The phosphorylated histone variant H2A.X (γH2A.X) is recognized for the formation of foci that serve as established markers of DNA double-strand breaks (DSBs). Nevertheless, the precise roles of H2A.X in the cellular response to genotoxic stress and the impact of the plant hormone abscisic acid (ABA) remain incompletely understood. In this investigation, we implemented CRISPR/Cas9 technology to produce loss-of-function mutants of *AtHTA3* and *AtHTA5* in *Arabidopsis*. The phenotypes of the *athta3* and *athta5* single mutants were nearly identical to those of the wild-type Col-0. Nevertheless, the *athta3 athta5* double mutants exhibited aberrant embryonic development, increased sensitivity to DNA damage, and higher sensitivity to ABA. The RT-qPCR analysis indicates that *AtHTA3* and *AtHTA5* negatively regulate the expression of *AtABI3*, a fundamental regulator in the ABA signaling pathway. Subsequent investigation demonstrated that *AtABI3* participates in the genotoxic stress response by influencing the expression of DNA damage response genes, such as *AtBRCA1*, *AtRAD51*, and *AtWEE1*. Our research offers new insights into the role of H2A.X in the genotoxic and ABA responses of *Arabidopsis*.

## 1. Introduction

Abiotic stress, which is the consequence of significant external environmental changes, can cause DNA damage, thereby jeopardizing the integrity and stability of the genome [1,2]. Various components of DNA, such as the purine and pyrimidine bases, sugar residues, and phosphodiester linkages, are susceptible to damage from both environmental and endogenous genotoxic agents [3]. A multitude of signaling pathways that are essential for DNA repair, cell cycle regulation, or cell death are initiated by exposure to genotoxins, DNA-damaging agents [4]. The coordination of repair processes at the loci of DNA damage, which are complex assemblies formed in the vicinity of the damaged site, is of the utmost importance [5,6]. In order to fit within the restricted nuclear space, the genomes of eukaryotic cells are condensed into chromatin fibers. The nucleosome is the fundamental component of chromatin. Each nucleosome is composed of approximately 150 base pairs of DNA that are wrapped around a histone octamer. This octamer is composed of two copies of the core histones H2A, H2B, H3, and H4 [7,8,9]. Distinctive histone variants have evolved in both animals and plants, playing critical roles in chromatin dynamics. The replication-independent incorporation of these variants can profoundly alter chromatin structure, influencing various biological processes such as transcriptional regulation and genome stability [10,11,12,13,14]. In mice, the essential role of histone variants in maintaining genomic stability is highlighted by the association of *H2A.X* loss with radiation sensitivity and growth retardation, both of which stem from compromised genomic stability [15]. In humans, H2A.X also participates in degradosome formation combined with endonuclease cyclophilin A and the apoptosis-inducing factor (AIF) enzyme to favor chromatinolysis processes under certain redox conditions within the cell [16,17]. The early cellular response to DNA damage is significantly influenced by the rapid phosphorylation of H2A.X at a conserved serine/glutamine (SQ) motif in its carboxyl terminus, which leads to the formation of γH2A.X [18,19,20,21,22,23]. Although the precise function of γH2A.X foci in yeast and mammals is a subject of debate, they are considered markers of DNA double-strand breaks (DSBs). The kinase activity of ataxia telangiectasia mutated (ATM) and Rad-3-related (ATR) kinase is the primary factor determining the rapid formation of γH2A.X foci following the induction of DSBs [24,25]. In *Arabidopsis*, there are two H2A.X isoforms, AtHTA3 and AtHTA5, which are distinguished by a mere two amino acids in their primary protein structure. Previous research has demonstrated that *H2A.X*-silenced lines, which employ RNA interference (RNAi) technology and retain residual gene expression, exhibit mild hypersensitivity to genotoxic agents, including bleomycin and camptothecin [26]. T-DNA insertional mutants for *H2A.X* demonstrate altered patterns of DSB repair, indicating that the H2A.X isoforms play both redundant and divergent roles in the modulation of Non-Homologous End Joining (NHEJ) and Homologous Recombination (HR) repair pathways [27]. It is important to note that the double mutants of *h2axa-2* (*athta5*) and *h2axb* (*athta3*) on a T-DNA background exhibited delayed radicle emergence in comparison to the wild-type Col-0. The *h2axb* mutants did not demonstrate a substantial difference in seed aging sensitivity compared to Col-0, whereas the *h2axa-2* mutants exhibited delayed germination [28].

Abscisic acid (ABA) is a critical plant hormone that controls a variety of biological processes, such as seed germination, plant growth, and responses to abiotic stress [29,30]. Studies have demonstrated that elevated levels of ABA inhibit DNA replication and cell division, which leads to growth retardation [31,32]. Major loss-of-function mutants of genes implicated in DSB repair, particularly in the NHEJ and HR pathways, demonstrate increased sensitivity to ABA treatment. An additional investigation of the phosphorylation status of H2A.X subsequent to ABA treatment has demonstrated that ABA induces γH2A.X and initiates a DNA damage response [33]. ABA activates HR-related genes during post-germination periods and stimulates the expression of DSB repair genes via HR during seed germination [33,34]. Abscisic acid insensitive 3 (ABI3), a transcription factor that is specific to plants and is a member of the B3 superfamily, is essential for the ABA signaling pathway and the transition from seed to seedling stages [35,36]. During seed maturation, the *abi3* mutants demonstrate severe defects, including an inability to degrade chlorophyll in dried seeds and an intolerance to desiccation [37,38,39]. The Sph/RY element CATGCA in the promoter region enables *ABI3* to activate its own expression at the transcriptional level [40]. It is induced by LEAFY COTYLEDON1 (LEC1), LEAFY COTYLEDON 2 (LEC2), and FUSCA3 (FUS3) [41,42,43]. Moreover, the transcriptional regulator DESPIERTO (DEP) and the chromatin-remodeling enzyme PICKLE (PLK) substantially reduce *ABI3* expression [44,45].

In this investigation, we effectively generated independent loss-of-function single mutants for *AtHTA3* and *AtHTA5* by utilizing clustered regularly interspaced short palindromic repeats/CRISPR-associated nuclease 9 (CRISPR/Cas9) technology, as well as a double mutant called *athta3 athta5* through crossing. Notably, the phenotypes of the *athta3* and *athta5* single mutants were indistinguishable from those of the wild-type Col-0. In contrast, the *athta3 athta5* double mutants exhibited a significant degree of ABA hypersensitivity, as well as apparent DNA damage and aberrant embryonic development. Our investigation demonstrated that the histone variant genes *AtHTA3* and *AtHTA5* were situated downstream of the ABA signaling regulatory gene *AtABI3*. Our results offer new perspectives on the function of AtH2A.X in the DNA damage-coupling ABA signaling pathway by influencing the expression of *AtABI3*.

## 2. Results

### 2.1. Phenotypes of athta3 and athta5 Loss-of-Function Mutants under Different DNA Damage Reagents

**Figure 1:**
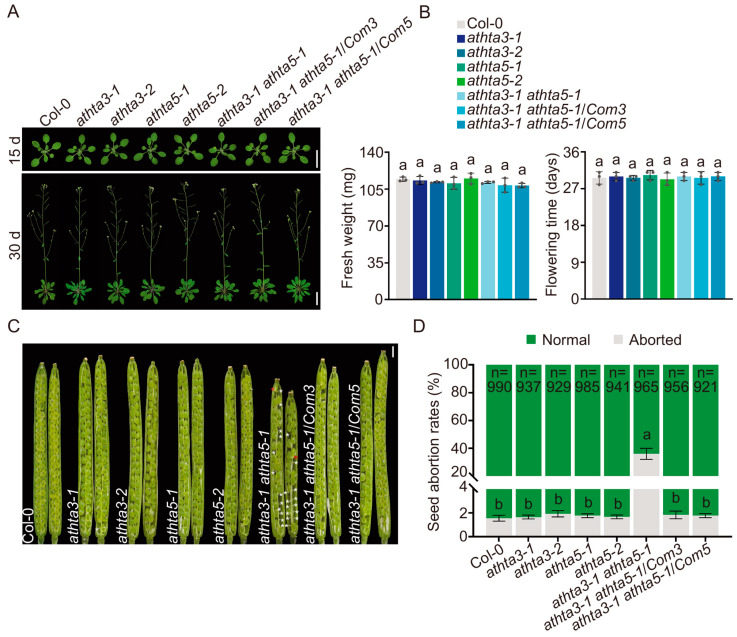
The phenotypes of Col-0, the *athta3* single mutants, the *athta5* single mutants, the *athta3 athta5* double mutants, and the *Com* lines grown under normal conditions. (**A**) Photographs of 15-day-old and 30-day-old plants grown under a long-day photoperiod (16 h light/8 h dark). White bars = 5 cm. (**B**) Measurement of fresh weight and flowering time for the indicated genotypes. The above-ground parts of 15-day-old plants were calculated as the fresh weight. The error bars represent the mean ± SD of three biological replicates, each with 15 plants. Lowercase letters denote the statistically significant differences among the groups (*p* < 0.05; one-way ANOVA, followed by Tukey’s multiple comparison test). (**C**) Photographs of longitudinal sections of siliques grown under a long-day photoperiod (16 h light/8 h dark). White bars = 1 mm. White asterisks indicate unfertilized ovules and red asterisks indicate aborted ovules. (**D**) Measurement of seed abortion rates for the indicated genotypes. Error bars represent the mean ± SD of three biological replicates, each comprising 15–20 siliques totally containing about 1000 seeds. Lowercase letters denote the significant differences among the groups (*p* < 0.05; one-way ANOVA, followed by Tukey’s multiple comparison test).

To investigate the function of *H2A.X* in *Arabidopsis*, we utilized CRISPR/Cas9 technology to generate independent mutants. Single-guide RNA (sgRNA) target sites for *AtHTA3* and *AtHTA5* were cloned into the CRISPR/Cas9 system driven by the *YAO* promoter [46]. In the *athta3* single mutants, a 1 bp insertion of either T or A at the position of 33 bp downstream of the start codon (ATG) resulted in a premature stop codon (Figure S1A). In the *athta5-1* mutants, a 1 bp insertion occurred at the position of 18 bp downstream of the ATG, while in the *athta5-2* mutants, a 7 bp deletion coupled with a 2 bp substitution was found at the position of 11 bp downstream of the ATG; both induced a frameshift mutation and consequently a premature stop codon (Figure S1B). We obtained *Cas9* gene-free plants by evaluating their resistance to hygromycin (Figure S1C). Subsequently, we crossed the *athta3-1* plants with the *athta5-1* plants to produce the *athta3-1 athta5-1* double mutants. We then established two complementation lines (*Com* lines) by expressing 2 × FLAG epitope-tagged *AtHTA3* or *AtHTA5* genomic DNA under the regulation of their native promoters into the *athta3-1 athta5-1* double mutant background. The expressions of the constructs *AtHTA3_pro_:gAtHTA3-2 × FLAG* and *AtHTA5_pro_:gAtHTA5-2 × FLAG* in the *Com* lines, designated as the *athta3-1 athta5-1*/*Com3* and the *athta3-1 athta5-1*/*Com5*, were confirmed using an anti-FLAG antibody (Figure S1D). During the vegetative stage, we observed that the *athta3* single mutants, the *athta5* single mutants, the *athta3 athta5* double mutants, and the two *Com* lines exhibited no abnormal phenotype and developed similar biomass (also defined as fresh weight) to the wild-type Col-0 (Figure 1A,B). Notably, in the *athta3-1 athta5-1* double mutants, we observed that silique sizes were smaller, and these siliques contained approximately 35% aborted seeds, contrasting sharply with the less than 2% seed abortion rate observed in the wild-type Col-0, the *athta3* and *athta5* single mutants, and the two *Com* lines (Figure 1C,D). Collectively, these findings suggested that *AtH2A.X* may have redundant functions in silique development and embryogenesis during the reproductive stages.

**Figure 2:**
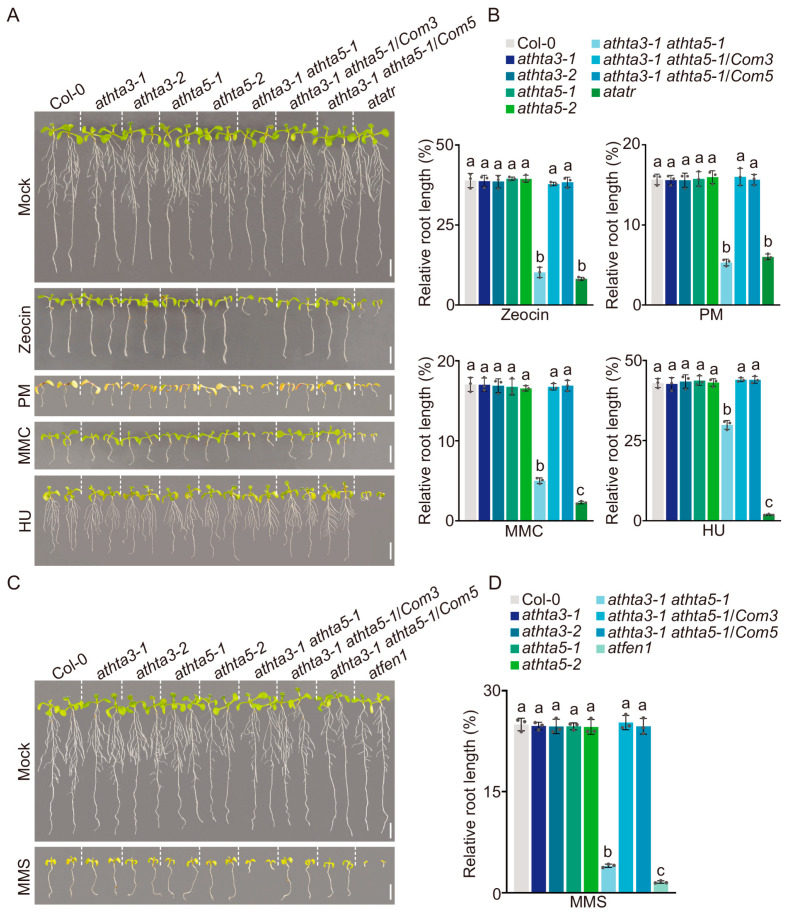
*AtHTA3* and *AtHTA5* play redundant roles in the response to DNA damage. (**A**,**C**) Sensitivity to genotoxic stress in the Col-0, the *athta3* single mutants, the *athta5* single mutants, the *athta3-1 athta5-1* double mutants, and the *Com* lines. The plants were grown in a liquid MS medium, which was supplemented separately with 25 μM Zeocin, 75 p.p.m. Phleomycin (PM), 10 μM mitomycin C (MMC), 1 mM hydroxyurea (HU), or 100 p.p.m. methyl methanesulfonate (MMS) for 14 days. The dash lines were used to distinguish different genotypes. White bars = 5 mm. The *atatr* or *atfen1* mutants were used as a control to assess DNA damage hypersensitivity. (**B**,**D**) Evaluation of the inhibitory effect of various DNA-damaging reagents on root growth. Relative root length was defined as the ratio of the root length under DNA damage conditions to the root length under control conditions. Error bars indicate the mean ± SD of three biological replicates, each with 20 seedlings. Lowercase letters represent the significant differences among groups (*p* < 0.05; one-way ANOVA, followed by Tukey’s multiple comparison test).

To evaluate the function of AtH2A.X in the response to DNA damage, reagents with different mechanisms were used: the DSB inducer Zeocin and Phleomycin (PM), the DNA intra-strand crosslinker mitomycin C (MMC), the DNA synthesis inhibitor hydroxyurea (HU), and the alkylating agent methyl methanesulfonate (MMS) [47]. DNA damage is recognized as triggering cell division arrest in the meristem [48]. Consequently, we measured the relative root length of the wild-type Col-0, the *athta3* single mutants, the *athta5* single mutants, and the *athta3-1 athta5-1* double mutants, as well as the *athta3-1 athta5-1*/*Com3* and the *athta3-1 athta5-1*/*Com5* lines (Figure 2). The *atatr* and *atfen1* mutants, known to be susceptible to DNA damage, were used as positive controls [49,50]. The *athta3-1 athta5-1* double mutants showed a much-decreased root development phenotype in the presence of the five chemicals mentioned above compared to the wild-type Col-0 and the *Com* lines, as shown in Figure 2. By contrast, there were no discernible phenotypes of root development in the *athta3* and *athta5* single mutants. These results suggested that *AtH2A.X* plays a major and redundant role in the DNA damage response.

To further substantiate the impact of *AtH2A.X* loss on the DNA damage response, we evaluated the expression profiles of critical DNA damage response genes in both the Col-0 and mutant lines. Our analysis encompassed genes that are recognized for their involvement in DNA damage repair, including *breast cancer susceptibility gene 1* (*AtBRCA1*) and *RAS associated with diabetes protein 51* (*AtRAD51*), which are genes involved in the HR repair pathway [51,52]. We also examined the expression of *TSO MEANING ‘UGLY’ IN CHINESE 2* (*AtTSO2*), which encodes a subunit of ribonucleotide reductase (RNR). This gene demonstrates transcriptional upregulation to mitigate the depletion of the nucleotide pool as a result of HU treatments [53]. Subsequently, we investigated the expression of *WEE1-like kinase* (*AtWEE1*). In response to replication stress induced by HU, *AtWEE1* induces cell cycle arrest [6,54]. As shown in Figure S2, we found that the expression levels of these DNA damage response genes were increased in the *athta3 athta5* double mutants, with the effect being particularly pronounced after exposure to genotoxic agents. These findings indicated that the loss of *AtH2A.X* function may reduce the efficacy of DNA damage repair, prolong the duration of cell cycle arrest, and elicit an enhanced DNA damage response.

### 2.2. Tissue-Specific Gene Expression Patterns and Subcellular Localizations of AtHTA3 and AtHTA5

**Figure 3:**
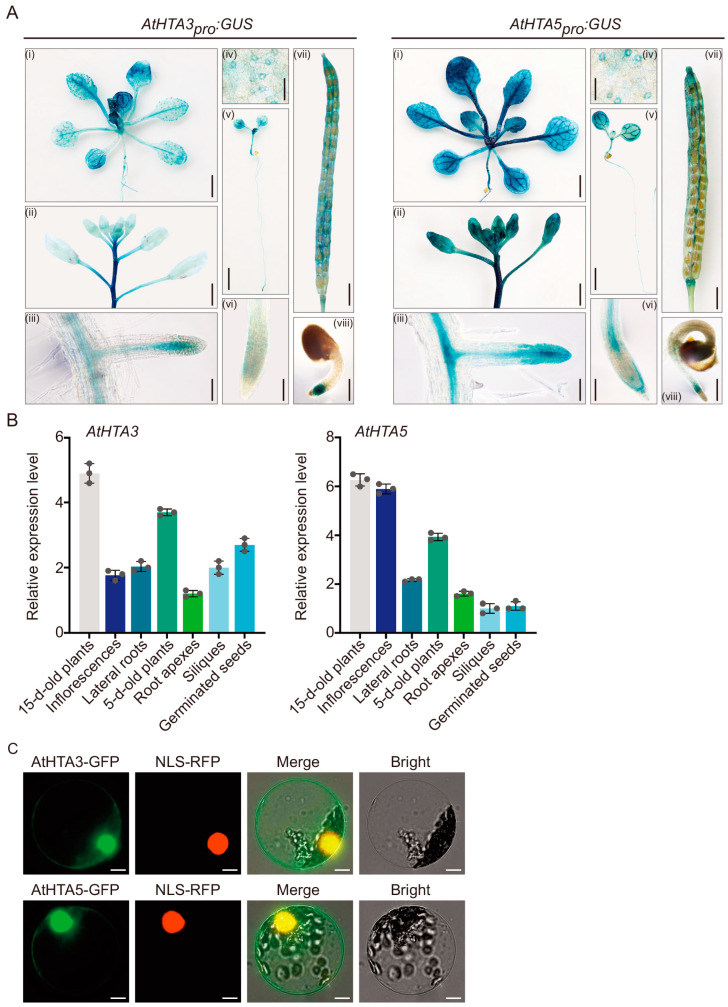
Tissue-specific expression patterns and subcellular localizations of *AtHTA3* and *AtHTA5*. (**A**) Transgenic plants expressing *AtHTA3_pro_:GUS* and *AtHTA5_pro_:GUS* were stained with X-Gluc at different stages and tissues, including 15 d old plants (i), inflorescences and stems (ii), lateral roots (iii), guard cells (iv), 5 d old plants (v), root apexes (vi), styles, valves, septums, ovule funiculi, abscission zones in mature siliques (vii), and germinated seeds (viii), to measurement expression patterns of *AtHTA3* and *AtHTA5*. Black bars = 3 cm (i); 2 cm (ii); 100 μm (iii); 5 mm (iv); 3 mm (v); 200 μm (vi); 1 cm (vii); 1 cm (viii). (**B**) Relative gene expression levels of *AtHTA3* or *AtHTA5* in the indicated tissues of Col-0 plants were determined by RT-qPCR, with *GAPDH* serving as the internal control. Data represent means ± SD of three biological replicates, each with indicated tissues from 10 to 20 plants for RNA extraction. Each circle on the plot is a replication of an independent experiment. Different colors indicate different tissues (**C**) Subcellular localizations of AtHTA3-GFP and AtHTA5-GFP. Protoplasts from Col-0 were co-transfected with constructs expressing AtHTA3-GFP or AtHTA5-GFP, as well as NLS-RFP as a nuclear marker. The fluorescence signals that resulted were analyzed with a confocal fluorescence microscope. GFP, green fluorescent protein; RFP, red fluorescent protein; Yellow signal: the overlap of green and red signals. White bars = 10 μm.

To investigate the expression patterns of *AtHTA3* and *AtHTA5*, whose N-terminal tails differ by only two amino acids, we generated transgenic plants that expressed the *β-glucuronidase* (*GUS*) reporter gene under the control of the native AtHTA3 and *AtHTA5* promoters (denoted by *AtHTA3_pro_:GUS* and *AtHTA5_pro_:GUS*). Various tissues, such as leaves, flowers, stems, lateral roots, root apexes, vascular tissues, guard cells, major veins of the cotyledons, styles, valves, septums, ovule funiculi and abscission zones in mature siliques, and germinated seeds, exhibited largely overlapping expression patterns for the two AtH2A.X isoforms (Figure 3A). Nevertheless, the *AtHTA5_pro_:GUS* construct exhibited substantially higher expression levels in roots and flowers than the *AtHTA3_pro_:GUS* construct. Furthermore, the stele, columella, and lateral root cap exhibited distinct *AtHTA5_pro_:GUS* activity, whereas *AtHTA3_pro_:GUS* exhibited only mild activity in these tissues (Figure 3A). Our findings were subsequently verified through reverse transcription-quantitative polymerase chain reaction (RT-qPCR) analysis. The *GUS* expression patterns were shown to be correlated with the transcript levels of *AtHTA3* and *AtHTA5*, as evidenced by the intensity of GUS staining in a variety of tissues (Figure 3B). To further examine the subcellular localization of AtHTA3 and AtHTA5, we generated fusion proteins by affixing green fluorescence protein (GFP) to the C-termini of AtHTA3 and AtHTA5, respectively. This process resulted in the recombinant proteins AtHTA3-GFP and AtHTA5-GFP. These constructs were subsequently introduced to the *Arabidopsis* protoplasts together with nuclear localization signal (NLS)-RFP (marker for labeling nucleus). Both AtHTA3-GFP and AtHTA5-GFP were primarily localized to the nucleus, as illustrated in Figure 3C. A minor fraction was also observed in the cytosol. Collectively, these findings suggested that *AtHTA3* and *AtHTA5* exhibited comparable spatial and temporal expression patterns.

### 2.3. AtHTA3 and AtHTA5 Function in the ABA Response

**Figure 4:**
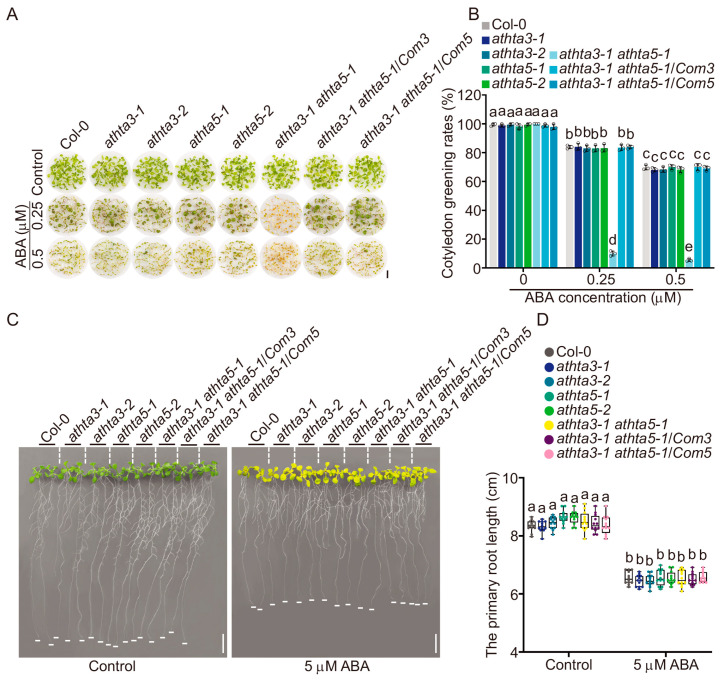
*AtHTA3* and *AtHTA5* function in the ABA response. (**A**,**B**) Measurement of cotyledon greening rates of Col-0, the *athta3* single mutants, the *athta5* single mutants, the *athta3 athta5* double mutants, and the *Com* lines. Plants were grown in liquid half-strength MS medium supplemented with DMSO, 0.25 μM or 0.5 μM ABA for 7 days. The cotyledon greening rate was the ratio of the number of seeds with cotyledon greening to the number of all cultivated seeds per genotype. Black bars = 5 mm. (**C**,**D**) Measurement of root length of Col-0, the *athta3* single mutants, the *athta5* single mutants, the *athta3 athta5* double mutants, and the *Com* lines. Plants were grown on half-strength MS medium for 4 days and then transferred to medium containing DMSO or 5 μM ABA for 14 days. The dash lines were used to distinguish different genotypes. The white lines at the bottom of each root show the approximate position of the root tip. White bars = 10 mm. Error bars indicate mean ± SD of three biological replicates, each with 50 seedlings in (**A**) and 12 seedlings in (**C**). Lowercase letters represent statistically significant differences (*p* < 0.05; two-way ANOVA, followed by Tukey’s multiple comparison test).

Prior research demonstrated that mutants associated with the HR pathway and DSB sensor genes exhibited relatively increased ABA sensitivities [33]. Therefore, we conducted a more thorough investigation of the ABA sensitivity of the *athta3* single mutants, the *athta5* single mutants, and the *athta3 athta5* double mutants in the presence of ABA during the seed germination and seedling growth stages. The phenotypic alterations were not detectable under normal conditions; however, the *athta3-1 athta5-1* double mutants exhibited an ABA hypersensitivity phenotype with reduced cotyledon greening rates (percentage of greening seedlings over all germinated seedlings) after germination on day 7 at varying concentrations of ABA (Figure 4A,B). Of note, the phenotypes of the *athta3-1 athta5-1*/*Com3* and the *athta3-1 athta5-1*/*Com5* lines were comparable to those of the *athta3* and *athta5* single mutants, as well as the wild-type Col-0 (Figure 4A,B). A high concentration of exogenous ABA inhibits root elongation [55,56], and to further investigate whether AtHTA3 and AtHTA5 are involved in the inhibition of root growth by ABA, the wild-type Col-0, the *athta3* single mutants, the *athta5* single mutants, the *athta3 athta5* double mutants, and the complementation lines were grown in solid medium containing 0 and 5 μM ABA. The primary root growth of the *athta3* and *athta5* single mutants, the *athta3-1 athta5-1* double mutants, the complementary lines and the wild-type Col-0 did not exhibit any phenotypic changes following ABA application during post-germination developmental phases (Figure 4C,D). These findings suggested that *AtHTA3* and *AtHTA5* function redundantly in response to ABA during the seed germination stage, but they are not engaged in the response to ABA in terms of primary root growth.

**Figure 5:**
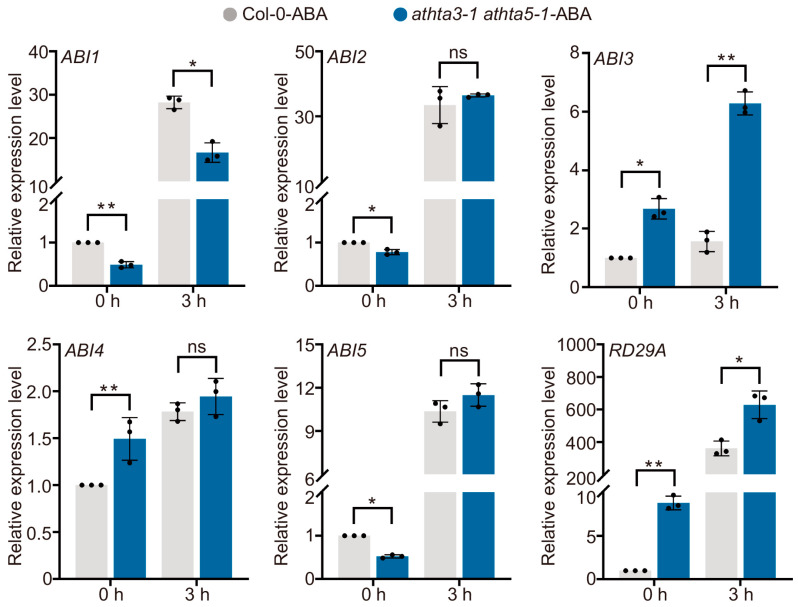
RT-qPCR validation analyses for validation of ABA-responsive gene expression. The 7-day-old Col-0 and *athta3 athta5* double mutants were treated under 10 μM ABA treatment for 0 h and 3 h. *GAPDH* was used as an internal control. Error bars indicate mean ± SD of three biological replicates. Each point on the plot indicated one independent replicate. Statistical analyses were performed by Student’s test (ns indicates not significant; * indicates *p* < 0.05; ** indicates *p* < 0.01).

To examine the possible role of AtHTA3 and AtHTA5 in response to ABA, we examine the expression of several important marker genes in ABA signaling using RT-qPCR analysis. It is intriguing that the expression levels of *Abscisic acid insensitive 1* (*AtABI1*) and *AtABI3* were the only ones that were substantially altered in the *athta3-1 athta5-1* double mutants in comparison to the wild-type Col-0 with or without ABA treatment (Figure 5). Previous research indicated that the cell cycle is a critical target for ABI3, and it is conceivable that the *abi3* mutation could result in ineffective ABA signaling in the cell cycle, thereby reducing seed quiescence [35]. Therefore, we proposed that the loss of function of *AtH2A.X* results in an abnormal embryo and an ABA-hypersensitive phenotype by excessively activating the expression of *AtABI3* under normal conditions and in response to ABA.

### 2.4. AtABI3 Functions Genetically Downstream of AtHTA3 and AtHTA5

**Figure 6:**
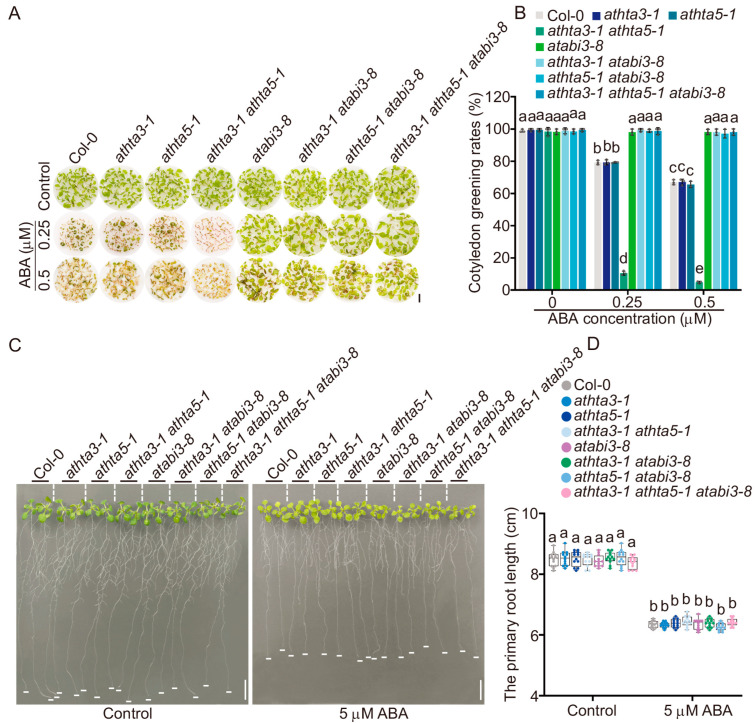
*AtABI3* acts genetically downstream of *AtHTA3* and *AtHTA5*. (**A**,**B**) Measurement of cotyledon greening rates of Col-0, the *athta3* single mutants, the *athta5* single mutants, the *athta3 athta5* double mutants, the *athta3 atabi3-8* double mutants, the *athta5 atabi3-8* double mutants, and the *athta3 athta5 atabi3-8* triple mutants. Plants were cultivated in liquid half-strength MS medium, which was supplemented with DMSO as a solvent and either 0.25 µM or 0.5 µM ABA for 7 days. The cotyledon greening rate was the ratio of the number of seeds with cotyledon greening to the number of all cultivated seeds per genotype. Black bars = 5 mm. (**C**,**D**) Measurement of root length for indicated genotypes. Plants were cultivated on half-strength MS medium for 4 days and then transferred to a medium containing DMSO or 5 µM ABA for 14 days. The dash lines were used to distinguish different genotypes. The white lines at the bottom of each root show the approximate position of the root tip. White bars = 10 mm. The error bars indicate the mean ± SD of three biological replicates, each with 50 seedlings in (**A**) and 12 seedlings in (**C**). The letters represent statistically significant differences among groups (*p* < 0.05; two-way ANOVA, followed by Tukey’s multiple comparison test).

Since AtHTA3 and AtHTA5 negatively impact the expression of *AtABI3*, we sought to examine the genetic interaction among *AtHTA3*, *AtHTA5*, and *AtABI3*. In order to produce the *athta3-1 atabi3-8* double mutants, the *athta5-1 atabi3-8* double mutants, and the *athta3-1 athta5-1 atabi3-8* triple mutants, we crossed the *athta3-1*, the *athta5-1*, and the *athta3-1 athta5-1* with *atabi3-8*, a loss-of-function mutant line that results in a critical amino acid conversion of leucine 298 to phenylalanine within the B1 domain [57,58]. In our assessment of ABA sensitivity following germination, the double mutants *athta3-1 atabi3-8* and *athta5-1 atabi3-8*, as well as the triple mutants *athta3-1 athta5-1 atabi3-8*, exhibited a hyposensitivity to ABA that was consistent with the *atabi3-8* single mutants during the germination phase (Figure 6A,B). Nevertheless, the *athta3-1 atabi3-8* double mutants, the *athta5-1 atabi3-8* double mutants, and the *athta3-1 athta5-1 atabi3-8* triple mutants did not exhibit phenotypes in terms of primary root growth after ABA treatment during seedling developmental phases (Figure 6C,D). This finding suggested that *AtABI3* functions genetically downstream of *AtHTA3* and *AtHTA5* during the germination stages of seeds that are inhibited by ABA.

### 2.5. AtABI3 Plays a Role in Genotoxic Stress Response

**Figure 7:**
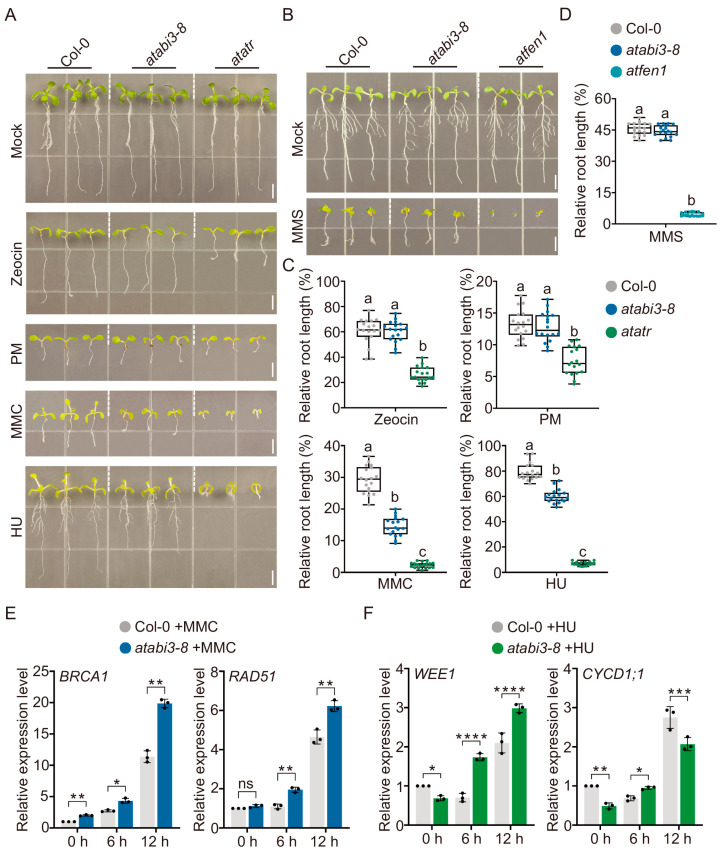
The involvement of *AtABI3* in the DNA damage response. (**A**,**B**) Sensitivity to genotoxic stress of Col-0 and *atabi3-8*. For a period of 14 days, the plants were cultivated in liquid MS medium and supplemented with variable concentrations of genotoxic reagents: 25 µM Zeocin, 75 p.p.m. Phleomycin (PM), 10 µM mitomycin C (MMC), 1 mM hydroxyurea (HU), or 100 p.p.m. methyl methanesulfonate (MMS) separately. The *atatr* and *atfen1* mutants were employed as controls for DNA damage hypersensitivity. The dash lines were used to distinguish different genotypes. White bars = 5 mm. (**C**,**D**) Root length measurement. Relative root length was defined as the ratio of the root length under DNA damage conditions to the root length under control conditions. The mean ± SD of three biological replicates with 18 seedlings each is denoted by the error bars. Significant differences among groups are indicated by lowercase letters (*p* < 0.05; one-way ANOVA, followed by Tukey’s multiple comparison test). (**E**,**F**) RT-qPCR validation analyses of DNA damage response-related marker genes. Col-0 and *atabi3-8* were treated under 10 μM MMC or 1 mM HU for 0 h, 6 h and 12 h. *GAPDH* was used as an internal control. Error bars indicate mean ± SD of three biological replicates. Each point on the plot indicated one independent replicate. Statistical analyses were performed by Student’s test (ns indicates not significant; * indicates *p* < 0.05; ** indicates *p* < 0.01; *** indicates *p* < 0.001; **** indicates *p* < 0.0001).

Given that the cell cycle is a likely target for ABI3, as previously reported [35], and considering that *AtABI3* expression is down-regulated by AtHTA3 and AtHTA5, we conducted further research to determine if AtABI3 is implicated in the DNA damage response. The *atabi3-8* mutants were subjected to various DNA-damaging agents, including Zeocin, PM, MMC, HU, and MMS, with the *atatr* and *atfen1* single mutants serving as positive controls. As illustrated in Figure 7A–D, the *atabi3-8* mutants displayed hypersensitivity to MMC and HU, as evidenced by stunted root growth and diminished leaf size relative to the wild-type Col-0. In contrast, other DNA-damaging agents had no significant impact on the growth of the *atabi3-8* mutants. These findings indicated that *AtABI3* plays a role in the response to genotoxic stress, particularly in the context of inter-strand crosslinks (ICLs) and replication fork stalling-induced DNA damage. To substantiate the impact of *AtABI3* loss on the DNA damage response, we assessed the expression levels of key DNA damage response genes, namely *AtBRCA1*, *AtRAD51*, and *AtWEE1*. The results demonstrated that the absence of *AtABI3* function resulted in a notable upregulation of these genes following MMC and HU treatments in comparison to the wild-type Col-0 (Figure 7E,F). This finding highlighted a disrupted DNA damage response. Moreover, we investigated the expression of *AtCYCD1;1*, which encodes a D-type cyclin that plays a crucial role in cell cycle re-entry during the activation of the meristem, thereby facilitating germination and early seedling growth [54,59]. In the *atabi3-8* mutants, *AtCYCD1;1* expression demonstrated fluctuating patterns under HU treatment in comparison to the wild-type Col-0 (Figure 7F). This variability may be attributed to a synchronized progression through mitosis induced by HU, which ultimately results in the observed root growth retardation [53].

## 3. Discussion

The previously published *H2A.X*-silenced line (*miH2AX*) retained 15% of *H2AXa* (*AtHTA5*) and 52% of *H2AXb* (*AtHTA3*) expression levels compared to the wild type. This partial retention of expression is likely the reason why the *miH2AX* line shows minimal phenotypic abnormalities and only a modest growth delay when exposed to agents that induce DSBs [26]. Notably, the *H2AXa;H2AXb* double mutants, with T-DNA insertion in the promoter region of *H2AXa* and intron region of *H2AXb*, also failed to completely abolish *H2A.X*. The *H2AXa;H2AXb* double mutants show no significant effect on fertility, only a marginal effect on root growth, even under conditions of ionizing radiation [60]. Previous studies have implicated *H2AXb* in both NHEJ and HR DNA repair mechanisms, whereas *H2AXa* appears to have a more pronounced influence on HR. This differential involvement suggests that the two isoforms may modulate different DSB repair pathways [27]. Therefore, establishing and studying the complete knockout (KO) lines is crucial for thoroughly elucidating the function of AtH2A.X.

In this study, leveraging the capabilities of CRISPR/Cas9 technology, we successfully generated several independent *AtHTA3* and *AtHTA5* loss-of-function mutants that differ from those in previous reports. Notably, we found that neither the *athta3* nor the *athta5* single mutants exhibited growth retardation under normal conditions or when treated with genotoxic agents. However, the *athta3 athta5* double mutants displayed heightened sensitivity to genotoxic stress induced by Zeocin, PM, MMC, HU, and MMS, as compared to the single mutants, the wild-type control (Col-0), and the complementation lines. The *h2axa-2 h2axb* double mutants exhibited substantial hypersensitivity to MMS, X-rays, and MMC, as previously noted by Wanda et al. [28]. Of note, they also observed that the spatial and temporal expression patterns of the two isoforms are primarily overlapping. This observation is comparable to ours. Previous studies have shown that Zeocin and PM, members of the bleomycin family, are known to induce DSBs [61]. MMC is recognized for causing inter-strand crosslinks, which are addressed through the γH2A.X-mediated Fanconi anemia (FA) repair pathway [62]. HU leads to replication fork stalling due to the depletion of the deoxyribonucleotide (dNTP) pool, thereby inhibiting DNA replication [63]. MMS is known for causing DNA base damage, which could be repaired through the ADP-ribosylation of H2A.X [64]. According to our findings, AtH2A.X is a critical component of numerous DNA repair pathways in *Arabidopsis*, and these two isoforms could complement each other’s functions.

We also identified that the ABA-hypersensitive phenotypes of *athta3 athta5* double mutants were observed during seed germination, as opposed to under seedling growth conditions. New insights into the interaction between the DNA repair pathway and ABA signaling are provided by the ABA overly sensitive mutant excessively ABA-sensitive mutant *abo4-1*, where *ABO4* encodes DNA pol ε, as evidenced by previous research. In *abo4-1*, ABA exposure results in an elevation of DSBs, genome instability, and an increase in HR, marked by the upregulation of DSB-responsive genes such as *MEIOTIC RECOMBINATION 11* (*MRE11*) and *GAMMA RESPONSE 1* (*GR1*), and the downregulation of others like *RAD51, BRCA1,* and *KU70* [34]. *Arabidopsis* DNA replication factor C1 (RFC1), a principal component of the RFC complex, is implicated in somatic HR and is subject to ABA modulation. ABA intervention leads to a decline in HR and the expression of genes pertinent to DNA damage response in *rfc1* mutants, underscoring *RFC1*’s role in ABA-mediated HR [65]. Furthermore, the *Arabidopsis* loss-of-function mutants for genes involved in the DSB sensing and signaling (*atatm-2*, *atatr*, *atmre11*, *atrad50* and *atnbs1*), NHEJ (*atku80*, *atku70*, *atpol*ε-*1*, *atlig4* and *atxrcc4*), and HR (*atrad51*, *atrad52*, *atrad54* and *atbrca1*) pathways have all exhibited ABA hypersensitivity [33]. These results emphasize the interaction between ABA responses and genotoxic stress. Intriguingly, we discovered that the expression of *AtABI3* is negatively regulated by AtHTA3 and AtHTA5 in response to ABA. The evidence that *AtABI3* expression is significantly induced in *athta3 athta5* mutants under ABA treatment corroborates this outcome.

The transition from a germinating embryo to an autotrophic seedling is hampered by dehydration and duress. A critical stress hormone, ABA, regulates the cell developmental checkpoints in plants that are experiencing water deficiency. AtABI3, a critical transcription factor, is responsible for the sustained but reversible growth arrest of germinated embryos in response to stress. As previously reported, the *abi3-1* mutant is incapable of executing the cell developmental checkpoints [66]. In our study, we unexpectedly found that another *AtABI3* loss-of-function mutant, *atabi3-8*, showed retarded growth under the treatment of MMC and HU, during seedling growth stages. We also found that, by RT-qPCR analysis, the expression patterns of a number of significant DNA damage response genes, including *AtBRCA1*, *AtRAD51*, and *AtWEE1*, were different from the wild-type Col-0. These data suggested that ABA may affect the genotoxic stress response through *AtABI3* and that AtABI3 may be involved in the regulation of these DNA damage-responsive genes.

## 4. Materials and Methods

### 4.1. Plant Materials and Growth Conditions

This study utilized *Arabidopsis thaliana* (L.) Heynh. ecotype Columbia-0 (Col-0) as the model plant system. Seedlings intended for crosses, developmental phenotype observations, and propagation were grown in soil under greenhouse conditions. In contrast, seedlings designated for stress treatments were germinated and cultivated on Murashige and Skoog (MS) medium [67] containing 1% sucrose (adjusted to pH 5.7 with KOH) within growth chambers. The plants were subjected to a 16 h light/8 h dark photoperiod at temperatures of 23 °C during the light phase with 160 μmol m^−2^ s^−1^ intensity and 21 °C during the dark phase, with a maintained relative humidity of 60%. The *atabi3-8* and *atatr* (*SALK_032841*) mutants were sourced from the Nottingham *Arabidopsis* Stock Centre (NASC), with homozygosity confirmed through PCR-based genotyping employing LB/RP or LP/RP primer sets (see Table S1 for details). The *atfen1* mutants were generously provided by Zhizhong Gong [50].

### 4.2. Plasmid Construction

CRISPR/Cas9 vectors were generated by designing guide targets for *AtHTA3* and *AtHTA5* using the online tool available at http://www.rgenome.net [68]. Subsequently, the single-guide RNA cassette was cloned into the *pYAO:hSpCas9* binary vector using the restriction enzyme *Spe*I [46]. To generate *AtHTA3_pro_:GUS* and *AtHTA5_pro_:GUS* constructs, a 2094 bp fragment upstream of the *AtHTA3* start codon and a 2200 bp fragment upstream of the *AtHTA5* start codon were individually amplified and cloned into the *pCAMBIA3301* binary vector using *Eco*RI and *Nco*I sites. The cDNA fragments of *AtHTA3* and *AtHTA5*, excluding stop codons, were amplified from the *Arabidopsis* leaf cDNA library and then individually inserted into the *326-sGFP* plasmid using the restriction sites *Xba*I and *Bam*HI sites. To construct *AtHTA3_pro_*:*AtHTA3-2* × *FLAG* and *AtHTA5_pro_*:*AtHTA5-2* × *FLAG* complementation plasmids, the promoter region along with genomic sequences lacking stop codons of *AtHTA3* and *AtHTA5* were inserted into the *pCAMBIA1302* binary vector using *Eco*RI and *Bam*HI sites through the In-Fusion HD Cloning Kit (Takara, 639650, Kusatsu, Japan). All primers used are listed in Table S1.

### 4.3. Mutant Isolation and Generation of Transgenic Plants

Transgenic *Arabidopsis* plants were generated using the *Agrobacterium tumefaciens*-mediated transformation method, employing the floral dip technique in the presence of 0.05% Silwet L-77 (Coolaber, CS9791, Shanghai, China) [69]. *Agrobacterium tumefaciens* GV3101 strain was cultivated on Luria–Bertani (LB) broth medium containing 50 mg/L rifampicin (Sangon Biotech, A430385, Shanghai, China). For the isolation of CRISPR/Cas9 mutants, T1 progeny seeds were selected on a B5 medium containing 50 mg/L hygromycin (Sangon Biotech, A600230, Shanghai, China). The presence of mutations in *AtHTA3* and *AtHTA5* was confirmed by Sanger sequencing. Homozygous T3 lines, which no longer contained the Cas9 construct, were chosen for further experimentation. For the construction of *athta3 athta5* double mutants by crossing, based on Mendel’s genetic law, F2 populations of plants (an average of 40 per F2 population) were screened for homozygosity or heterozygosity for the mutation of *AtHTA3* or *AtHTA5* locus genotyped using HTA3-CR-Check and HTA5-CR-Check primer sets, and separated homozygous F3 seeds were used for further experiments. For complementation lines, T1 generation seeds were screened on B5 medium containing 50 mg/L hygromycin, and resistant seedlings were planted in soil, T2 populations with approximately 75% resistant seedlings, indicating single-locus T-DNA insertion, were considered for further analyses with confirmation using anti-FLAG antibody (Sigma, F3165, Setagaya, Japan), and homozygous T3 seeds were used for further experiments. For promoter-GUS transgenic plants, T1 seeds were selected on B5 medium containing 0.2‰ Basta (Sangon Biotech, A614229, Shanghai, China), and resistant seedlings were planted in soil with confirmation using GUS staining. T2 populations with single-locus T-DNA insertion were considered for further analyses.

### 4.4. DNA Damage Agents and ABA Treatments

For DNA damage sensitivity assays, sterilized seeds were cultured in liquid MS medium with various concentrations of DNA damage agents: 25 μM Zeocin (Invitrogen, R250-01, Waltham, MA, USA); 75 p.p.m. Phleomycin (Sangon Biotech, A620211-0100, Shanghai, China); 10 µM MMC (Selleck, S8146, Houston, TX, USA); 1 mM HU (Sigma, H8627, Burlington, MA, USA); 100 p.p.m. MMS (Sigma, 129925, Burlington, MA, USA). The medium was refreshed every 2 days to ensure chemical efficacy. After 2 weeks, seedlings were transferred to a solid MS medium for measurement and imaging. For ABA sensitivity assays for monitoring cotyledon greening, sterilized seeds were sown in liquid ½ MS medium with 0, 0.25, or 0.5 µM ABA (Coolaber, CA1011, Beijing, China). The percentage of greening was determined after 7 days, with images taken for documentation. To evaluate root growth, 4-day-old seedlings with similar initial root lengths were transplanted to solid ½ MS medium with 0 or 5 µM ABA and allowed to grow vertically. Subsequently, the root lengths were reassessed after a further 2 weeks of growth.

### 4.5. GUS Staining and Subcellular Localization

For the GUS staining experiment, transgenic plants *Arabidopsis* harboring either the *AtHTA3_pro_:GUS* or *AtHTA5_pro_:GUS* constructs were infiltrated with a GUS staining solution (Clontech, 631721, Kusatsu, Japan) and incubated at 37 °C. Following the staining procedure, the plants were subjected to an overnight clearing process. To observe the subcellular localization of AtHTA3 and AtHTA5, mesophyll protoplasts isolated from 3-week-old seedlings were co-transfected with *AtHTA3-GFP* or *AtHTA5-GFP* along with *NLS-RFP* constructs using polyethylene glycol (PEG)-mediated transfection [70]. Once the transfection was complete, the protoplasts (2 × 10^5^ mL^−1^) were incubated in 1 mL W5 buffer (pH 5.7) containing 2 mM MES, 5 mM KCl, 154 mM NaCl, and 125 mM CaCl_2_ at 22 °C for 12 to 16 h in a growth chamber and then visualized using the fluorescence microscope (Olympus, BX53, Tokyo, Japan).

### 4.6. RT-qPCR Analysis

Briefly, total RNA was isolated using TRIzol reagent (Invitrogen, 15596026CN, Waltham, MA, USA), and RNA quality was assessed by detecting the A260/A280 absorption ratio using a spectrophotometer (Thermo Fisher Scientific, Waltham, MA, USA), followed by formaldehyde agarose gel electrophoresis to evaluate RNA integrity. cDNA was synthesized from 2 μg total RNA using the One-Step gDNA Removal and cDNA Synthesis SuperMix Kit (TransGen Biotech, AT311-03, Beijing, China). Each reaction included 25 ng cDNA with PowerUp SYBR Green Master Mix (Thermo Fisher Scientific, A25742). The length of the qPCR products is 80-200 bp. The cycling conditions were set in the QuantStudio™ 5 Real-Time PCR System (Applied Biosystems, Carlsbad, CA, USA) according to the manual, with an initial hold stage at 50 °C 2 min and 95 °C 2 min, followed by 95 °C 15 sec for denaturation and 60 °C 1 min for annealing/extension with 40 cycles in the PCR stage. Melt curve analysis was performed to ensure specific amplification. QuantStudio™ Design and Analysis Software v1.2.x (Applied Biosystems, Carlsbad, CA, USA) was used to acquire raw qPCR data. The 2^−ΔΔCt^ method was used for data analysis [71], and the expression stability of *GAPDH* as an internal control was assessed using BestKeeper software (https://www.gene-quantification.de/bestkeeper.html) [72]. All reactions were performed using samples from three independent biological replicates. All primers used for RT-qPCR are listed in Table S1.

### 4.7. Statistical Analysis

All experimental data were expressed as the means ± standard errors (SD). Normality of the data was assessed using the Shapiro–Wilk normality test with R v.4.0.1 software. One- or two-way ANOVA statistical analyses were performed using GraphPad Prism v.9.0 software.

## 5. Conclusions

In conclusion, our investigation demonstrates that the histone variants AtHTA3 and AtHTA5 play crucial roles in DNA damage repair and ABA-mediated seed germination, possibly by regulating the ABA signaling gene *AtABI3*. This study lays a foundation for further research on the role of AtH2A.X in the DNA damage-coupling ABA signaling pathway by influencing the expression of *AtABI3* in *Arabidopsis*. Further investigations are necessary to ascertain if AtABI3 can directly interact with the signaling molecules involved in DNA damage response.

## Data Availability

All data and materials presented in this paper are available on request from the corresponding author.

## Supplementary Materials

The following supporting information can be downloaded at https://www.mdpi.com/article/10.3390/ijms25168940/s1.
