# Supplementary material for: Arabidopsis Histone Variant H2A.X Functions in the DNA Damage-Coupling Abscisic Acid Signaling Pathway
Source: Int J Mol Sci. 2024 Aug 16;25(16):8940. doi: 10.3390/ijms25168940 (PMC11354415; doi:10.3390/ijms25168940)
Supplement: Supplementary file 1 [file ijms-25-08940-s001.zip › ijms-3102924-supplementary.pdf]

Supplementary materials

Table S1 The list of primers used in this study

Primer name	Sequence (5′–3′)	Used for
HTA3-CDS	F: ATGAGTTCCGGCGCCG<br>R: TTAAAACTCTTGAGAAGCAGATCCG	Gene cloning
HTA5-CDS	F: ATGAGTACAGGCGCAGGAA<br>R: TCAGAACTCCTGAGAAGCAGAT	Gene cloning
HTA3-Promoter	F: gactcttgaccatggCGAGGGTGAAGGACCCATT<br>R: tcagatctaccatggTTTTTGTTTATCGGGAAATTTGA	GUS staining
HTA5-promoter	F: gactcttgaccatggTGCACCTAGTCATAGATTGAAAA<br>R: tcagatctaccatggCGTCTTCTTCGAAATTTAGAACA	GUS staining
HTA3-Com	F: catgattacgaattcCGAGGGTGAAGGACCCATT<br>R: gtcgtccttgtagtcAAACTCTTGAGAAGCAGATCCG	Complementation lines
HTA5-Com	F: catgattacgaattcTGCACCTAGTCATAGATTGAAAA<br>R: gtcgtccttgtagtcGAACTCCTGAGAAGCAGAT	Complementation lines
HTA3-GFP	F: acgggggactctagaATGAGTTCCGGCGCCG<br>R: gctcaccatggatccAAACTCTTGAGAAGCAGATCCG	Subcellular localization
HTA5-GFP	F: acgggggactctagaATGAGTACAGGCGCAGGAA<br>R: gctcaccatggatccGAACTCCTGAGAAGCAGAT	Subcellular localization
HTA3-CR-Target	F: attgGCAGTGGAACAACTAAAGG<br>R: aaacCCTTTAGTTGTTCCACTGC	CRISPR/Cas9
HTA5-CR-Target	F: attgATGAGTACAGGCGCAGGAAG<br>R: aaacCTTCCTGCGCCTGTACTCAT	CRISPR/Cas9
HTA3-CR-Check	F: CACTCACAAAATCCTCAGCCATC<br>R: CTTCAAACCGAATTCATTACTGGA	Mutation sequencing
HTA5-CR-Check	F: AACAAATCACAATCAATCTTAGTAGCC<br>R: TCAGTTCCATTGATACATGATTCGT	Mutation sequencing
Cas9-Check	F: CCACTCCATCAAGAAGAACCTC<br>R: TTGGACTTGAAGTTAGGGGT	Cas9-free isolation
atr-LP<br>*atr*-RP<br>LBb1.3	F: GCAGCAAAAATTTCTTGGTTG<br>R: ACTTCAAGGGTTCCGATGTTC<br>ATTTTGCCGATTTCGGAAC	T-DNA genotyping
HTA3-qPCR	F: CTCACAAAATCCTCAGCCATCTC<br>R: CTTTCCTCTGCCACCTTTAGTTG	RT-qPCR
HTA5-qPCR	F: AACAAATCACAATCAATCTTAGTAGCC<br>R: CTGCGCCTGTACTCATCGTCT	RT-qPCR
BRCA1-qPCR	F: GTTACGTGTGCAAAACTCATACCAGAATG<br>R: GATACTTGTTTAGGCTGAGAGTGCAGTGG	RT-qPCR

RAD51-qPCR	F: CTCCGAGGAAGGATCTCTTGCAG<br>R: GCTCGCACTAGTGAACCCCAGAGG	RT-qPCR
WEE1-qPCR	F: TCATCGGCGTTTCAGGGT<br>R: CTAAGCCGCTCATCAAGTTCTG	RT-qPCR
TSO2-qPCR	F: GAACGAATCATCGCTTTCGCTTGC<br>R: CGTCACGTGAGATCAATTCGTTTGAG	RT-qPCR
CYCD1;1-qPCR	F: CCTGGTGATTCTATCGCTTGTTTTA<br>R: CGCTTGTACCTTGAGAATCCATG	RT-qPCR
ABI1-qPCR	F: CGTCTCACATCTTCGTCGCT<br>R: TCAATCCTCGCAGCTTCATC	RT-qPCR
ABI2-qPCR	F: CGGTTCTCAGGTAGCGAATTATT<br>R: AGCCTTCTTCCACTTCTCTTGC	RT-qPCR
ABI3-qPCR	F: TTTGGTTGCGGCGAAGG<br>R: GGTGTCAAAGAACTCGTTGCTATC	RT-qPCR
ABI4-qPCR	F: CGTTAGGGCAGGAACAAGGA<br>R: GGATCCAGACCCATAGAACATACC	RT-qPCR
ABI5-qPCR	F: ATGGGAGGGCTAAGGGGAA<br>R: ACTCCGCCAATGCATGTTTTA	RT-qPCR
RD29A-qPCR	F: ACACACCAGCAGCACCCAGA<br>R: TGATCTTGCTCATGCTCATTGC	RT-qPCR
GAPDH-qPCR	F: TTGGTGACAACAGGTCAAGCA<br>R: AAACTTGTCGCTCAATGCAATC	RT-qPCR

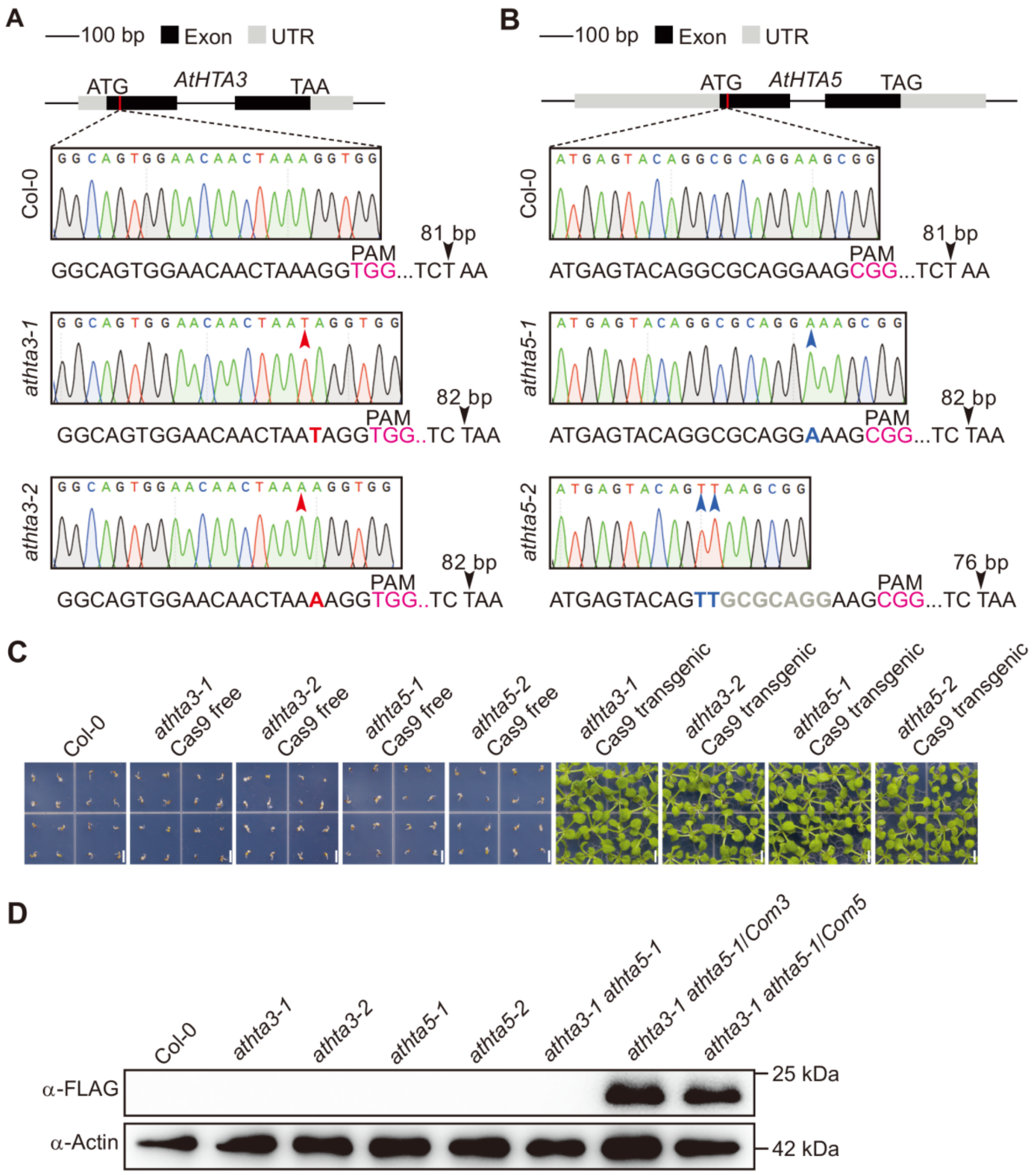

Figure S1. Generation of *ath2a.x* mutants and complementation lines. **(A and B)** Sequence chromatograms of Sanger sequencing showing the mutations of *AtHTA3* or *AtHTA5* generated by CRISPR/Cas9 technology. Red bar: target site. Red and blue arrows indicats the mutation sites. Protospacer adjacent motif (PAM) sites are highlighted in magenta. **(C)** Isolation of *Cas9*-free mutants. Different genotypes were grown on solid media supplemented with hygromycin (50 mg/L). Photos were taken 2 weeks after sowing. White bars = 5 mm. **(D)** Immunoblot analysis of *AtHTA3$_{pro}$:gAtHTA3-2×FLAG* and *AtHTA5$_{pro}$:gAtHTA5-2×FLAG* in complementation lines using anti-FLAG antibody. Actin2 was used as a loading control.

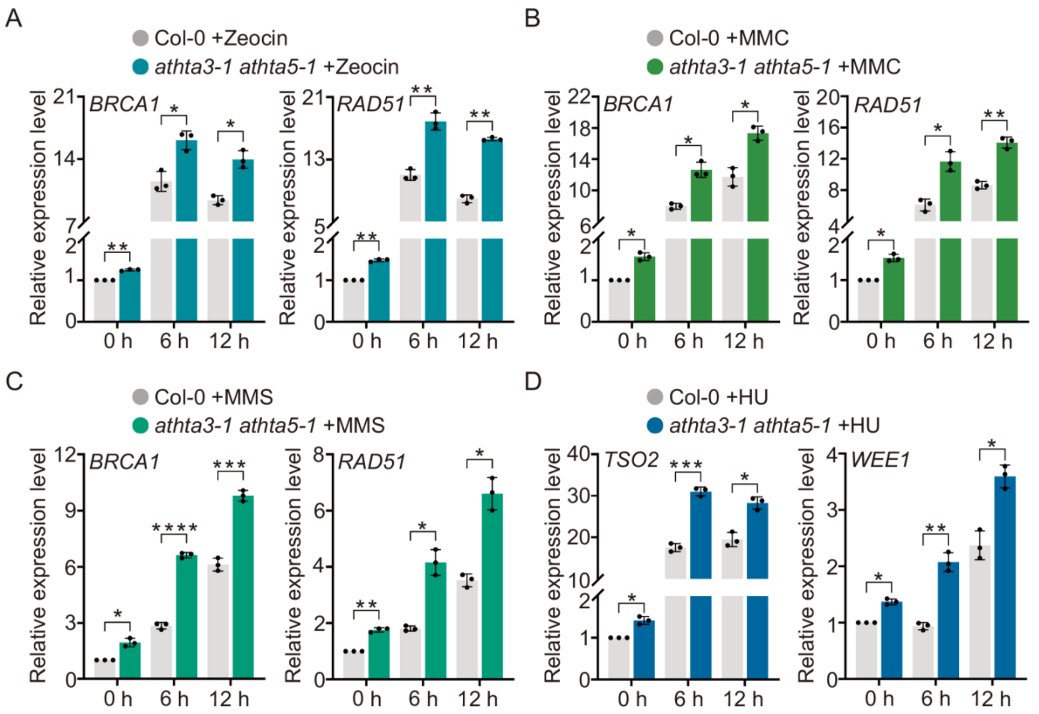

Figure S2. RT-qPCR validation analyses of DNA damage response-related marker genes. (**A-D**) The wild-type Col-0 and the *athta3-1 athta5-1* double mutants were separately treated under 25 µM Zeocin, 10 µM mitomycin C (MMC), 100 p.p.m. methyl methanesulfonate (MMS), or 1 mM hydroxyurea (HU) for 0 h, 6 h and 12 h. *GAPDH* was used as an internal control. Error bars indicate mean ± SD of three biological replicates, each with 20 plants. Each black point on the plot indicated one independent replicate. Statistical analyses were performed by Student's test (* indicates $p < 0.05$; ** indicates $p < 0.01$; *** indicates $p < 0.001$; **** indicates $p < 0.0001$).
